# Psychotherapy Under Lockdown: The Use and Experience of Teleconsultation by Psychotherapists During the First Wave of the COVID-19 Pandemic

**DOI:** 10.32872/cpe.6821

**Published:** 2022-09-30

**Authors:** Jessica Notermans, Pierre Philippot

**Affiliations:** 1 Consultations Psychologiques Spécialisées, Université Catholique de Louvain, Louvain-La-Neuve, Belgium; 2 Laboratory for Experimental Psychopathology, Université Catholique de Louvain, Louvain-La-Neuve, Belgium; University of Vienna, Vienna, Austria

**Keywords:** teleconsultation, COVID-19, attitudes, online psychotherapy, ethics, therapeutic relationship

## Abstract

**Background:**

Facing the COVID-19 pandemic, some psychotherapists had to propose remote consultations, i.e., teleconsultation. While some evidence suggests positive outcomes from teleconsultation, professionals still hold negative beliefs towards it. Additionally, no rigorous and integrative practice framework for teleconsultation has yet been developed. This article aims to explore the use and experience of teleconsultation by 1) investigating differences between psychotherapists proposing and not proposing it; 2) evaluating the impact of negative attitudes towards teleconsultation on various variables; 3) determining the perceived detrimental effect of teleconsultation, as opposed to in-person, on the therapeutic relationship and personal experience; and 4) providing insights for the development of a teleconsultation practice framework.

**Method:**

An online survey was distributed via different professional organisations across several countries to 246 (195 women) French-speaking psychotherapists.

**Results:**

Psychotherapists who did not propose teleconsultation believed it to be more technically challenging than psychotherapists who proposed it, but felt less constrained to propose it, and had less colleagues offering it. Attitudes towards teleconsultation showed no significant associations with therapeutic relationship, personal experience, and percentage of teleconsultation. As compared to in-person, empathy, congruence, and therapeutic alliance were perceived to significantly deteriorate online, whereas work organisation was perceived to be significantly better. While most psychotherapists proposed remote consultations, they did not provide adaptations to such setting (e.g., ascertaining a neutral video background); nor used videoconferencing platforms meeting privacy and confidentiality criteria.

**Conclusion:**

Training and evidenced-based information should be urgently provided to practitioners to develop rigorous guidelines and an ethically and legally safe practice framework.

## Highlights

- Psychotherapists differ in their perceptions of teleconsultation as whether they propose it or not.
- Attitudes towards teleconsultation are not related to its use nor to the therapeutic relationship.
- Teleconsultation worsens perceived therapeutic relationship, but improves work organisation.
- Training is needed to improve an ethically and legally safe practice of teleconsultation.

Following the first wave of the COVID-19 pandemic, many countries imposed a lockdown, which resulted in the suspension of various healthcare practices, including face-to-face psychotherapy. Consequently, many psychotherapists had to rapidly adapt their services and propose consultations at a distance, i.e., teleconsultation. Teleconsultation refers to “interactions that happen between a clinician and a client for the purpose of providing diagnostic or therapeutic advice through electronic means” (Pan American Health Organization, 2021). This drastic change in the provision of mental health services was largely improvised as most psychotherapists and professional organisations were unprepared for this challenge.

Some evidence suggests positive outcomes from teleconsultation for the treatment of specific conditions (Acierno et al., 2016; Poletti et al., 2021; Wright & Caudill, 2020). Moreover, recent evidence from the COVID-19 pandemic also shows that most psychotherapists experience remote psychotherapy rather positively (Feijt et al., 2020; Humer et al., 2020; McBeath et al., 2020). These attitudes towards teleconsultation are influenced by a set of factors (Connolly et al., 2020), such as previous online experience, clinical experience (Békés & Aafjes-van Doorn, 2020), perceived ability to develop a strong therapeutic relationship (Aafjes-van Doorn et al., 2021; Roesler, 2017), and perceived therapeutic efficacy (Aafjes-van Doorn et al., 2021). In contrast, other evidence reports that mental health practitioners hold negative attitudes towards teleconsultation (Mendes-Santos et al., 2020; Perle et al., 2013; Varker et al., 2019). Beliefs regarding poor efficacy (Schulze et al., 2019) and ethical limitations (Stoll et al., 2020) of such practices may hamper its use and implementation, as well as reduce clients’ adhesion. Questions regarding the strengths and limitations of online therapy are known topics of discussion among mental health professionals (Rochlen et al., 2004). Therefore, it is important to further investigate current attitudes towards teleconsultation and evaluate their potential impact.

Last but not least, with the drastic transition from in-person to remote consultation, several authors underlined the importance to develop an integrative and balanced practice framework with specific guidelines to inform psychotherapists about the use of teleconsultation (Smith et al., 2020). Boldrini and colleagues (2020) provided a set of recommendations to help professionals support the implementation and use of teleconsultation. Moreover, another team of researchers listed useful evidence-based guidelines for clinicians using telepsychiatry (Smith et al., 2020). However, these recommendations are gathered from country-specific sources (Italy and England respectively), and thus do not allow for a global perspective on the matter. Finally, while a set of valuable recommendations regarding the policy and practice of telepsychotherapy was also developed in field studies (Shore et al., 2018; Van Daele et al., 2020), and suggested by professional organisations (British Association for Behavioral & Cognitive Psychotherapies, 2021), they are largely based on clinical consensus. Further empirical data are thus required to provide a rigorous, ethical, and safe framework to support the provision of remote mental healthcare in times of crisis (Ohannessian et al., 2020).

In this perspective, the present survey aims to explore the use and experience of teleconsultation among French-speaking psychotherapists in order to provide insights regarding its challenges and benefits. First, we hypothesise that there will be significant differences between psychotherapists proposing teleconsultation and those who do not, specifically in terms of attitudes towards it, previous online experience, feelings of constraint, perceived support, and colleagues’ usage. Second, attitudes towards teleconsultation will have significant and negative associations with the therapeutic relationship, the personal experience of teleconsultation, and the percentage of teleconsultation proposed. Third, the therapeutic relationship and personal experience of teleconsultation will be perceived as significantly worse than in-person. Lastly, this study will explore how various elements of teleconsultations (e.g., legal and ethical questions, adaptations, etc.) may contribute to the elaboration of a practice framework. Altogether, it investigates the information, skills, and knowledge that would help psychotherapists improve their practice of teleconsultation, in terms of effectiveness, ethics, and well-being at work. Thus, it may serve as a basis for establishing psychotherapists’ potential needs for training in teleconsultation, as suggested by recent studies (Van Daele et al., 2020; Wijesooriya et al., 2020).

## Method

### Recruitment and Procedure

The survey was developed online (on the Qualtrics platform), and distributed via different professional organisations (e.g. UPPCF, AEMTC) to 246 French-speaking psychotherapists between September 15^th^ and October 31^st^ of 2020 in Belgium, France, Morocco, Switzerland, and Tunisia. The study was approved by the IPSY Ethics Committee of UCLouvain (Project 2020-30; approved on June 10^th^, 2020).

### Survey Questionnaire

The questionnaire (Appendix 1, Supplementary Materials) comprises four sections. Section 1 presents the aim of the study and provides informed consent details. If consent was given, participants were asked whether they proposed teleconsultations from the first lockdown (March 16^th^, 2020) onwards. Those who answered positively were directed to Section 2; others were directed to Section 3.

Section 2 includes questions pertaining to the use and experience of teleconsultation for psychotherapists proposing it. Section 3 examines the attitudes towards teleconsultation of psychotherapists not proposing it, as well as other variables that may shed light on the motives behind their non-adhesion to teleconsultation. Section 4, was given to all participants, and covers demographics, namely gender, level of education, level of psychotherapy training, psychotherapeutic orientation, work status, years of experience, percentage of teleconsultations proposed since June 2020, living situation, number of dependent children and their age, if any, the extent to which the charge of dependent children living at home impacted their psychotherapy activities during the lockdown, age, and country of residence.

### Measures

To the authors’ knowledge, no valid and reliable measures evaluating their questions of interest were found in the literature. Therefore, the survey’s validity and reliability are limited. Survey’s questions are detailed below (see Appendix 1 in the Supplementary Materials for the full survey).

Section 2 contains 20 questions inquiring on: 1) whether the number of consultations in 2020 decreased or increased (ranging from -100 to +100%) between March and June, and 2) between July and September, as compared to the same period in 2019; 3) attitudes (i.e., negative beliefs) towards teleconsultation, evaluated on a 5-point Likert scale ranging from 0 “Strongly disagree” to 4 “Strongly agree”, from an 11-item ad hoc questionnaire; 4) what remote mediums were utilised (telephone, chat messaging, e-mails, and/or videoconferencing); 5) the type of platforms used (e.g., Zoom, Whatsapp, Whereby, etc.); 6) whether they had prior experience with teleconsultation (No experience; Experience as a supervisee/or as a patient; Experience as a supervisor; and/or Experience as a psychotherapist); 7) whether they felt constrained to use it (Not at all, Slightly, Moderately, or Strongly); 8) whether their colleagues used it (None; A few, Some, Most, or All); 9) whether they had specific concerns regarding data protection and confidentiality (No; “Yes, I found satisfactory answers”; or “Yes, but I still have questions (specify)”); 10) whether they received support to set up teleconsultation (No, Mild, Moderate, or Complete support); 11) whether (Yes or No), and 12) how they encouraged clients to engage in teleconsultation (selecting from a 10-item ad hoc questionnaire items such as “providing information regarding the efficacy of teleconsultation”, “providing a short free trial on the media used”, etc.); 13) whether they provided adaptations to the teleconsultation setting (“Generally, I did not have to adapt the teleconsultation setting” or “I had to do minor changes”), and 14) how they adapted their online interventions, based on the population (e.g., children, adolescents, adults, etc.), and 15) disorder (e.g., mood disorder(s), anxiety disorder(s), eating disorder(s), etc.). Question 16 investigated the percentage of clients for whom their issue was directly linked to the pandemic, aggravated by it, or independent from it. Question 17 evaluated, on a 5-point Likert scale (from 0 “Highly degraded” to 4 “Highly improved”), psychotherapists’ experience of teleconsultation as compared to in-person for the therapeutic relationship (empathy, congruence, positive regard, and therapeutic alliance). Question 18 asked whether psychotherapists will continue to propose teleconsultation after the pandemic (“Yes, based on the patient/client demand, teleconsultation will be an option”; “Yes, teleconsultation will become major in my clinical practice”; or No). Question 19, evaluated on a 5-point Likert scale (from 0 “Much worse” to 4 “Much better”), psychotherapists’ personal experience of teleconsultation (therapeutic efficacy, professional satisfaction, fatigue/exhaustion, work organisation, and ease of payment) as opposed to in-person. A final open-ended question asked about additional comments/remarks regarding teleconsultation.

Section 3 includes seven questions. First, a 7-item ad hoc questionnaire evaluates on a 5-point Likert scale (from 0 “Not at all important” to 4 “Very important) psychotherapists’ motives for not providing teleconsultation (e.g., “this mode of communication does not seem appropriate for a psychotherapy”, or “people did not wish to start/continue via teleconsultation”). Then, participants were asked whether they could have received support if they proposed teleconsultation (No, Mild, Moderate, or Complete support); whether they felt constrained to offer it (Not at all, Slightly, Moderately, or Strongly); whether they had previous experience with it (No experience; Experience as a supervisee/or as a patient; Experience as a supervisor; and/or Experience as a psychotherapist); whether their colleagues were offering it (None, a Few, Some, Most, or All); and whether they intended to propose it in the future (No; “Yes, if the pandemic persists”; or “Yes, no matter what”). Finally, the same ad-hoc questionnaire from section 2 investigated their attitudes towards teleconsultation.

### Data Analysis

For Hypothesis 1, an exploratory factor analysis (EFA) explored the internal structure of attitudes. The Kaiser–Meyer–Olkin index and the Bartlett sphericity test were computed to assess the robustness of the results. Then, independent *t*-tests evaluated the significant differences in attitudes between psychotherapists who did and did not propose teleconsultation, as well as for previous experiences, feelings of constraint, perceived support, and colleagues’ usage. Levene's corrections were used for cases in which variances differed between groups. For Hypothesis 2, Pearson’s correlations were calculated between attitudes towards teleconsultation, therapeutic relationship, personal experience, and percentage of teleconsultation. For Hypothesis 3, single sample *t*-tests determined whether teleconsultations were perceived as worse than in-person, for the therapeutic relationship and personal experience. Finally, for Hypothesis 4, single sample *t*-tests and descriptive statistics explored variables related to the use and experience of teleconsultation, and participants’ demographics. Qualitative data complemented quantitative results. IBM SPSS Statistics for Windows, Version 21.0, was used for all analyses.

## Results

### Participants Characteristics

A total of 246 individuals (195 women; 35 men) participated in the study. 16 participants did not fill the entire survey, mainly on demographic questions. They were aged between 25 and 70 years (*M* = 42.4). Out of 230 participants, all were psychotherapists and most of them (186) had at least 3 years of postgraduate training in psychotherapy. They were mostly from Belgium (133), Switzerland (45), and France (37). The majority were self-employed (114) or part-time self-employed (59), while 94 were employees. Most participants identified themselves as CBT (156) or integrative (58) psychotherapists. The majority (158) lived as a couple and 115 had children living at home (average 1.90 children). Dependent children living at home were aged between 0 and 29 years of age (*M* = 11.32). Out of the total sample (*N* = 246), 222 psychotherapists proposed teleconsultation (173 females; 33 males; 16 did not answer), and 24 (22 females; 2 males) did not.

### Hypothesis 1

The factorability of the 11 attitudes towards teleconsultation was examined for the total sample. A three-factor solution explained 58.2% of the variance for the entire set of variables, with eigenvalues greater than 1 and a minimum of 10% of variance explained by each factor. The Kaiser–Meyer–Olkin index for sampling quality was good: 0.191, and the Bartlett sphericity test was correct, χ^2^(55) = 75.84, *p* > .04. The scree plot also suggests a three-factor solution. The factor solution, after Oblimin rotation, is displayed in Table 1. The first factor, “attention”, pertains to the belief that teleconsultation entails attention difficulties in both the client/patient and therapist. The second factor, “technical issues”, covers beliefs that teleconsultation requires significant technical skills and infrastructure. The last factor, “interpersonal communication”, reflects the belief that teleconsultation is detrimental to the communication quality between client/patient and therapist.

**Table 1:** Factor Loadings, After Oblimin Rotation, for the 11 Items of Attitudes Towards Teleconsultation for Psychotherapists Proposing and not Proposing it

Items of attitudes towards teleconsultation	Factors		
	(1) Attention	(2) Technical Issues	(3) Interpersonal Communication (reversed)
I will be too distracted	.883		
I will not be engaged/present enough	.851		
The client/patient will not be engaged/present enough	.647		
There will be too many distractions in the individual	.572		
My personal infrastructure will not be adequate for teleconsultation (e.g., limited infrastructure, isolated room for the session, etc.)	.387	.328	
Teleconsultation requires a good handling of informatics tools		.838	
Technical issues will have too big of an impact on communication		.729	
The lack of non-verbal information will be too important			-.854
Teleconsultation will limit the development of a good therapeutic relationship			-.778
It will be difficult to set up some interventions		.433	-.505
Teleconsultation will increase dropout number in certain individuals (e.g., addictions)			-.357

*t*-tests were run to determine whether attitudes differed between psychotherapists as a function of whether they proposed teleconsultations. A significant difference was observed only for Factor 2, “technical issues”, indicating that psychotherapists who did not propose teleconsultations believed they entailed more technical issues, *t*(32.247) = -3.159, *p* = .003, than those who proposed them. Mean differences for each attitude towards teleconsultation between psychotherapists proposing and not proposing teleconsultations are found in Appendix 2, Supplementary Materials.

Most psychotherapists had no experience with teleconsultation before the pandemic, whether they proposed it (*n* = 188) or not (*n* = 19), with no difference between these two groups, χ^2^(1, *N* = 243) = 0.764, *p* = .382. Most psychotherapists who proposed teleconsultation felt significantly more constrained (i.e., Strongly constrained; *M* = 2.78, *SD* = 1.17) to do so than those who did not (i.e., Slightly constrained; *M* = 1.79, *SD* = .66), *t*(41.249) = 6.340, *p* < .001. In addition, psychotherapists reported having received little support to set up teleconsultation, whether they offered it (*M* = 1.69, *SD* = .88) or not (*M* = 1.75, *SD* = 1.07), *t*(240)= -.321, *p* = .749. Qualitative data indicate that support mainly came from colleagues (*n* = 55), IT services (*n* = 22), supervisors (*n* = 13), friends and family (*n* = 15), and professional associations (*n* = 11). Finally, the majority of psychotherapists (*n* = 116) expressed that some colleagues used teleconsultation. Yet, psychotherapists proposing teleconsultation reported that most of their colleagues used it (*M* = 3.24, *SD* = .87) as opposed to colleagues of psychotherapists not offering it (*M* = 2.38, *SD* = .77), *t*(241) = 4.677, *p* < .001.

### Hypothesis 2

No significant correlations above the coefficient .30 were found. However, for exploratory purposes, significant (*p* < .001) and positive associations were found between therapeutic relationship, personal experience, and percentage of teleconsultation (Appendix 3, Supplementary Materials).

Regarding the therapeutic relationship, empathy is correlated with congruence (*r* = .389), unconditional positive regard (*r* = .411), therapeutic alliance (*r* = .417), therapeutic efficacy (*r* = .378), and professional satisfaction (*r* = .303). Thus, the more psychotherapists perceived empathy as better online than in-person, the more the above variables were perceived similarly, and vice versa. Comparably, congruence is correlated with therapeutic alliance (*r* = .394), and therapeutic efficacy (*r* = .413), while unconditional positive regard is only correlated with therapeutic alliance (*r* = .309). Finally, therapeutic alliance is correlated with therapeutic efficacy (*r* = .472), and professional satisfaction (*r* = .382).

Regarding therapeutic experience, therapeutic efficacy is correlated with professional satisfaction (*r* = .592), such that the more psychotherapists perceived therapeutic efficacy as better online than in-person, the more they perceived professional satisfaction as better online than in-person, and vice versa. Therapeutic efficacy is also correlated with percentage of consultation (*r* = .343), meaning that the more psychotherapists perceived therapeutic efficacy as better online than in-person, the more their percentage of teleconsultation increased from June 2020, and vice versa. Similarly, professional satisfaction is correlated with strain (*r* = .438), efficiency in work organisation (*r* = .352), and percentage of consultation (*r* = .356). Finally, strain is also correlated with efficiency in work organisation (*r* = .403).

### Hypothesis 3

Single sample *t*-tests against 3 (neutral “no change” point) showed significant changes in three aspects of the therapeutic relationship: empathy, congruence, and therapeutic alliance. Specifically, participants perceived that these significantly degraded online as compared to in-person (Table 2).

**Table 2:** Perceived Effect of Teleconsultation on Therapeutic Relationship as Compared to Face-To-Face (n = 207) (1: highly degraded, 3: no change, 5: highly improved)

Aspects of therapeutic relationship	*M*	*SD*	*p*
Empathy	2.81	0.59	< .001
Congruence	2.76	0.67	< .001
Unconditional Positive Regard	2.96	0.51	.206
Therapeutic Alliance	2.86	0.70	.006

Similarly, single sample *t*-tests against 3 showed that all variables of personal experience of teleconsultation were perceived as significantly worse online, as compared to in-person, except ‘Organisation, time and task management, etc.’ which was perceived as significantly better (Table 3).

**Table 3:** Experience of Teleconsultation as Compared to Face-to-Face (n = 206) (1: much worse; 3: no difference, 5: much better)

Variables of personal experience of teleconsultation	*M*	*SD*	*p*
Organisation, time and task management, etc.	3.24	1.17	.004
Ease/Rapidity to receive payments	2.49	0.91	< .001
Therapeutic Efficacy	2.39	0.77	< .001
Professional Satisfaction	2.24	0.96	< .001
Strain, Fatigue	2.24	1.12	< .001

### Hypothesis 4

#### Data From Psychotherapists Proposing Teleconsultation

During the first lockdown (from March to June 2020), consultations significantly dropped by almost 24% (*SD* = 46.03), *t*(221) = -7.759, *p* < .001 (single sample *t*-test against 0). This decrease was observed for all job status: self-employed (*n* = 110, *M* = -24.81, *SD* = 58.46); employees (*n* = 83, *M* = -20.34, *SD* = 42.31); and part-time self-employed (*n* = 47; *M* = -40.28, *SD* = 44.71). However, from June to September 2020, consultations appeared to have slightly but significantly increased by 6.3%, (*SD* = 31.68), *t*(220) = 2.945, *p* = .004, as compared to the same period in 2019. Such increase is also observed in all status: self-employed (*n* = 109, *M* = 2.95, *SD* = 30.09); employees (*n* = 83, *M* = 10.72, *SD* = 30.39); and part-time self-employed (*n* = 47, *M* = 11.57, *SD* = 39.56). Then, from June to September 2020, 19.1% of consultations, on average, occurred remotely.

Out of 115 participants with dependent children at home, 53.9% did not report a decrease in their professional activities. However, 20.0% slightly reduced (10.0% to 30.0%) their professional activities, 14.8% moderately reduced (31.0 to 60.0%), 6.1% strongly reduced (61.0 to 80.0%), and 5.2% extremely reduced them (81.0 to 100.0%). No significant gender difference was found; such that dependent children did not present more difficulties in professional activities for men, and vice versa.

Participants reported that the majority of their clients (*n* = 207, *M* = 64.9%, *SD* = 24.40) consulted for reasons independent of the COVID-19 crisis. 12.8% (*n* = 207, *SD* = 15.56) consulted for issues mainly related to COVID-19, and 29.5% (*n* = 207, *SD* = 21.23) consulted for issues significantly aggravated by COVID-19.

Out of 222 participants, 94.1% used videoconference for teleconsultations; 67.1% used the telephone; 12.6% used e-mails; and 4.5% used chat messaging. Regarding videoconferencing platforms, 63.6% of psychotherapists reported using Skype, 42.6% used Zoom, 29.2% used Whatsapp, and 26.3% used Whereby (Appendix 4, Supplementary Materials).

More than half of the participants (*n* = 116, 52.3%) found satisfactory answers regarding data protection and deontology issues, whereas a third (*n* = 61, 27.5%) did not have questions regarding these issues. Still, a fifth (*n* = 41, 18.9%) found answers but had remaining questions, mainly concerning the confidentiality of videoconferencing platforms (*n* = 32).

The majority of practitioners (*n* = 119, 54.6%) set up actions to encourage clients’ adhesion (Table 4). Most participants (*n* = 128, 57.7%) did not adapt their practice to the teleconsultation setting, while 27% (*n* = 60) provided minor changes. Some (*n* = 19, 8.6%) adapted their therapeutic procedures (e.g., screen sharing to show schemas or other visuals; printing materials to present before the camera; emailing questionnaires and other documents); others (*n* = 18, 8.1%) adapted their room, desk, and/or video background; and 12 participants (5.4%) adapted their schedules and/or consultation timing and frequency. A small portion of respondents, 12.6% (*n* = 28), adapted significantly their consultation based on the type of population (see Appendix 5, Supplementary Materials), while 10.4% (*n* = 23) adapted significantly their consultation based on the type of disorder (see Appendix 5, Supplementary Materials). Changes pertained mainly to therapeutic procedures and interventions (e.g., shortened session, flexible schedule, adaptation of interventions, etc.).

**Table 4:** Actions Set up to Encourage Adherence to Teleconsultation

Actions to encourage adherence to teleconsultation	*N* = 222	%
Communicating with the patient/client to assess the situation (via email, telephone, or other)	97	43.7
Giving general advice to ensure optimal conditions for teleconsultations (e.g., be in a quiet space to avoid distractions and increase privacy, ensure a good internet connection, have charged devices, etc.)	79	35.6
Underlining the importance of psychotherapy continuity for the well-being of the patient/client	75	33.8
Giving information on the use of virtual platform (or other used media)	69	31.1
Being flexible regarding schedule	67	30.2
Giving information on the privacy of personal data (confidentiality regarding the session and the used media)	51	23
Doing a trial test on the used media	45	20.3
Giving information on the efficacy of teleconsultations	41	18.5
Being flexible regarding payments	29	13.1

Finally, out of 207 respondents, 65.8% (*n* = 146) intend to keep teleconsultation as an option, after the lockdown, if requested by their client. Only, 6.8% (*n* = 15) intend to rely mainly on teleconsultation in their clinical practice. In contrast, 20.7% (*n* = 46) intend to not use teleconsultation anymore after the lockdown.

Qualitative results from participants’ comments (*n* = 74) provided additional useful information. Some participants (*n* = 12) underlined numerous advantages (e.g., facility to consult regardless of geographical distance, schedule flexibility), while others (*n* = 17) enumerated disadvantages and difficulties (e.g., increased fatigue, lack of warmth, difficulty to set up specific intervention and/or share therapeutic information). Few (*n* = 5) underlined that there was no important difference between teleconsultation and in-person. Some (*n* = 4) were agreeably surprised by teleconsultation and saw their attitudes improved after using it. Finally, 12 participants explained that the majority of clients refused to pursue via teleconsultation.

#### Data From Psychotherapists not Proposing Teleconsultation

Only 24 participants did not propose teleconsultation during the lockdown. The two main reasons behind this decision concerned personal issues, and the belief that this type of communication was not appropriate for psychotherapy (Table 5). Open answers showed that personal reasons (*n* = 8) pertained mainly to limited infrastructure (*n* = 6), such as having access to adequate IT material or a private room. More than half (*n* = 14; 58.3%) do not have the intention to use teleconsultation in the near future; over a third (*n* = 9; 37.5%) will use it if the pandemic persists; and one participant definitely intends to use it in a near future.

**Table 5:** Reasons for Not Proposing Teleconsultation (from 1: not at all important to 5: very important)

Reasons for not proposing teleconsultation	*M*	*SD*
Personal reasons (e.g., limited infrastructure, childcare, etc.)	3.75	1.62
This type of communication does not seem appropriate for psychotherapy	3.54	1.10
Individuals did not want to start or pursue via teleconsultation	3.54	1.38
Lack of IT support	3.46	1.44
I have doubts regarding the therapeutic efficacy in teleconsultation	3.13	1.23
The (mental) state of individuals did not require the continuity of therapy	2.42	1.21
Financial reasons (e.g., to receive governmental or other financial aid)	1.75	1.11

## Discussion

This survey shows that most psychotherapists rapidly responded to the sanitary crisis by proposing teleconsultations. They did so with little support and no previous experience with teleconsultation. The important drop (24%) in consultations observed during the first lockdown might have fostered for some the rapid transition to teleconsultation.

Regarding the first hypothesis, while psychotherapists who did not propose teleconsultation believed it to be more technically challenging, received less support, and had less colleagues using it, than those proposing it, attitudes towards teleconsultation did not appear to significantly influence its use. Similar findings from a recent systematic review (Connolly et al., 2020) suggest that, overall, practitioners tend to have positive attitudes towards telemental health regardless of its disadvantages. Moreover, they suggest that previous experience as well as repetitive use of telemental health is related to positive attitudes and acceptance of such method. Comparably, our qualitative data suggest that most therapists felt reassured about these issues after gaining some experience with teleconsultation, and surprisingly pleased; a finding also expressed in Elford et al.’s study (2000). Additionally, qualitative data suggest that the main determinant for not proposing teleconsultation lied in contextual factors, rather than being a personal choice. For example, working in an institution (e.g., hospital, prison) or at home made it difficult to set up teleconsultations due to the lack of appropriate infrastructure (e.g., IT material, stable internet connection, private room). Connolly et al. (2020) describe similar negative attitudes regarding the disadvantages of telepsychiatry but underline that the benefits of such methods often outweigh its costs. Nevertheless, it is important to note that the sample of psychotherapists not proposing teleconsultation in the present survey is rather small, which calls for caution in interpreting the findings.

Rejecting our second hypothesis, no significant correlations were evidenced between attitudes and teleconsultation’s use and experience. A similar finding was reported by Monthuy-Blanc and colleagues (2013), such that intention to use telepsychotherapy was not determined by providers’ attitudes towards it, neither by how difficult they expected it to be, but merely by how useful they thought it to be to First Nations clients in Australia. Nevertheless, a recent study also reported that therapists’ concerns about online connectedness predicted negative attitudes towards teleconsultation and decreased perceived efficacy (Békés et al., 2021). Therefore, it would be of interest to pursue researching the impact of attitudes on the experience of teleconsultation.

In accordance to our third hypothesis, most aspects of the therapeutic relationship (empathy, congruence, and therapeutic alliance) were perceived as significantly deteriorated online, as compared to in-person, with the exception of unconditional positive regard. Moreover, participants also reported that their personal experience with teleconsultation in terms of ease of payment, work exhaustion, therapeutic efficacy, and professional satisfaction was also perceived as significantly worse online. Unexpectedly, however, work organisation was perceived as significantly better online.

Regarding our fourth hypothesis, a plethora of findings could be used to help in the development of a practice framework. First, privacy and confidentiality information and trainings should be urgently provided to professionals. In fact, in our survey, the majority of platforms used (e.g., Skype, Whatsapp, Messenger) does not reach the minimal legal criteria for privacy and confidentiality (e.g., some platforms record and sell communication data) as requested by psychotherapy. Moreover, ethical concerns are raised by the fact that many respondents (27.5%) did not seem concerned about deontology and data protection issues with respect to teleconsultation. However, current guidelines and recommendations from different countries strongly underline the importance of ensuring the privacy and confidentiality of videoconferencing platforms (American Psychological Association, 2020; British Association for Behavioral & Cognitive Psychotherapies, 2021; Commission des Psychologues, 2020; Shore et al., 2018; Smith et al., 2020; Van Daele et al., 2020). As Lustgarten et al. (2020) explain, even if some platforms (e.g., Skype, FaceTime) may be familiar for most providers and clients, other platforms may be more secure and legally compliant. These authors also provide further recommendations regarding safe practice. Evidently, guidelines and recommendations must be made more accessible to all psychotherapists, and professional organisations should work actively in providing recommendations and safe-to-use platforms and apps protecting clients’ personal information (Ohannessian et al., 2020).

Second, psychotherapists should keep encouraging clients’ adhesion to teleconsultation. In the survey, half of the psychotherapists proposing teleconsultation actively sought to motivate their clients to accept teleconsultation. They mostly kept in touch with them and provided information regarding its use, safety, and efficacy. In fact, showing informational videos discussing the benefits of internet-based mental health services increases clients’ acceptance (Ebert et al., 2015). Surprisingly, however, only 20% proposed a trial on the chosen media, while theory and anecdotal evidence suggest this action to be very effective (Sasangohar et al., 2020; Smith et al., 2020).

Third, information and training should be provided regarding contextual and therapeutic adaptions to the teleconsultation setting. In the survey, most therapists did not significantly adapt their way of delivering psychotherapy beyond the switch towards teleconsultation. However, it is important to have a proper and professional setting for teleconsultation (British Association for Behavioral & Cognitive Psychotherapies, 2021; De Witte et al., 2021; Sasangohar et al., 2020; Smith et al., 2020), such as ensuring that their video background conveys a feeling of safety and intimacy, and ensuring that clients are benefiting from a quiet, secure, and undisrupted space for the therapy session. More importantly, therapists should be aware of their clients’ location in order to contact them in case of communication failure (e.g., having a contact cell phone number) or emotional breakdown (e.g., having a backup person in the client’s immediate surrounding who could be reached and intervene). Regarding interventions, only slight adaptations were provided. Our qualitative data and anecdotal evidence suggest that many therapists avoid interventions entailing the activation of intense or aversive emotions, such as exposure. However, recent evidence suggests that such interventions can be successfully and safely provided online (Wells et al., 2020). Furthermore, few adaptations were reported as a function of clients’ age or disorder, although some authors (Smith et al., 2020; Van Daele et al., 2020) emphasise that teleconsultation be adapted to the population, its context, and the conditions they are facing. Other authors and clinicians provide recommendations on how to adapt therapeutic interventions to the teleconsultation setting for groups (Banbury et al., 2018), children (American Academy of Children and Adolescent Psychiatry, 2021; American Psychiatric Association, 2020; Becqueriaux, 2020; Landrum, 2020), as well as for people suffering from eating disorders (Waller et al., 2020) and post-traumatic stress (Kaltenbach et al., 2021; Moring et al., 2020). Nevertheless, such works are still in their infancy and more empirical evidence is needed to optimise the provision of teleconsultation.

The present survey suffers from some limitations. First, it has been conducted online and among French-speaking psychotherapists, thus reducing its reach to participants from other countries, and with minimal Internet literacy and/or accessibility. Second, from a lack of valid measurements in the literature, no psychometrically sound measures could be used to evaluate our hypotheses. Third, a memory bias may have impacted our findings, as psychotherapists were asked retrospectively about their use and experience of teleconsultation. Finally, it should also be noted that while this survey addressed the first lockdown, the situation kept evolving. Further surveys, targeting the following phases of the pandemic should examine these evolutions in terms of increase in the provision of teleconsultation and professionals’ exhaustion.

### Conclusion

While some findings enlightened the use and experience of teleconsultation by psychotherapists during the first lockdown, many questions remain in all discussed domains: the impact of attitudes towards the use and experience of teleconsultation; the legal and ethical aspects of videoconferencing platforms; and ways to develop contextual and therapeutic adaptations to the teleconsultation setting. It is the authors’ opinion that basic psychotherapy training should address these questions, and that professional organisations should provide detailed information and instructions about the use of ethically and legally safe teleconsultation platforms.

## Supplementary Materials

The Supplementary Materials include the entire survey questionnaire and additional tables related to some results (e.g., mean differences, correlations, etc.). For access see Index of Supplementary Materials below.

10.23668/psycharchives.8181 Supplement 1 Supplementary materials to "Psychotherapy under lockdown: The use and experience of teleconsultation by psychotherapists during the firstwave of the COVID-19 pandemic"

NotermansJ.
PhilippotP.
 (2022). Supplementary materials to "Psychotherapy under lockdown: The use and experience of teleconsultation by psychotherapists during the first wave of the COVID-19 pandemic"
[Survey questionnaire, and additional tables]. PsychOpen. 10.23668/psycharchives.8181
PMC966733536398003

## References

[sp1_r1] Notermans J. Philippot P. (2022). Supplementary materials to "Psychotherapy under lockdown: The use and experience of teleconsultation by psychotherapists during the first wave of the COVID-19 pandemic" [Survey questionnaire, and additional tables]. PsychOpen. 10.23668/psycharchives.8181 PMC9667335 36398003

