# Supplementary material for: Psychotherapy Under Lockdown: The Use and Experience of Teleconsultation by Psychotherapists During the First Wave of the COVID-19 Pandemic
Source: Clin Psychol Eur. 2022 Sep 30;4(3):e6821. doi: 10.32872/cpe.6821 (PMC9667335; doi:10.32872/cpe.6821)
Supplement: Supplement 1 [file cpe-04-6821-s01.pdf]

Psychotherapy Under Lockdown: The Use and Experience of Teleconsultation by Psychotherapists During the First Wave of the COVID-19 Pandemic

Jessica Notermans, Pierre Philippot

Clinical Psychology in Europe
https://doi.org/10.32872/cpe.6821

Appendices

Appendix 1. Survey questionnaire

Enquête Téléconsultation Thérapeute

Start of Block: Présentation

INTRO

Évaluation de la téléconsultation chez les psychothérapeutes dans le contexte de la pandémie du Coronavirus COVID-19

OBJECTIFS GENERAL DU PROJET

Vous êtes invité·e à participer à une enquête en ligne visant à récolter des informations sur votre utilisation et expérience de la téléconsultation (en comparaison au face-à-face) en tant que psychologue et dans le contexte de la pandémie du Coronavirus COVID-19. Au terme de cette étude, notre but ultime est de pouvoir améliorer ce mode de consultation et promouvoir les meilleures pratiques dans ce contexte particulier. La complétion ne devrait pas dépasser **15 minutes**. Votre participation se fait sur une **base volontaire** et est entièrement **anonyme**. Vous êtes libre d'interrompre votre participation à tout moment sans que cela n'engendre de conséquences et sans qu'il soit nécessaire de justifier votre décision. Il se peut que vous, et d'autres personnes, puissent bénéficier de cette étude car elle aidera à informer la pratique clinique sur les besoins, questions et préoccupations concernant la téléconsultation. Les résultats de cette recherche pourraient être utilisés dans un but scientifique et pourraient être publiés. L'anonymat et la confidentialité des données sont garantis dans chaque partie de cette étude en accord avec le règlement national, incluant le Règlement Général sur la Protection des Données (RGPD). Nous vous invitons à parcourir les informations ci-dessous relevant des objectifs et implications de l'étude afin que vous puissiez prendre une décision réfléchie et informée quant à votre participation. Veuillez lire le texte de consentement éclairé et cocher votre accord de participation (dans la case prévue à cet effet). Après avoir donné votre consentement, l'enquête en ligne débutera.

CONTACT Pour toute question, préoccupation, et/ou besoin d'information supplémentaire ou si vous considérez avoir subi

un préjudice lié à cette étude, les investigateurs Jessica Notermans (jessica.notermans@student.uclouvain.be) et Pr. Pierre Philippot (pierre.philippot@uclouvain.be) restent disponibles.

○ Si vous souhaitez plus d'information sur les conditions de participation de cette étude, cliquez ici.

Display This Question:

If Évaluation de la téléconsultation chez les psychothérapeutes dans le contexte de la pandémie du C... = Si vous souhaitez plus d'information sur les conditions de participation de cette étude, cliquez ici.

INFOS

INFORMATIONS ESSENTIELLES À VOTRE DÉCISION DE PARTICIPER **Objectifs et description du protocole de l'enquête** Cette enquête investigue diverses questions relatives à vos représentations de la téléconsultation ; aux changements perçus et/ou mis en place (ou non) lors de votre transition vers la téléconsultation ; ainsi qu'à votre expérience générale de cette transition. Au terme de cette enquête, le but ultime est donc de pouvoir améliorer ce mode de consultation et de promouvoir les meilleures pratiques dans ce contexte particulier. Pour participer, il faut être majeur et avoir le français comme langue maternelle (ou avoir un niveau suffisant de compréhension et d'écriture). **Déroulement de l'étude** Avant de commencer l'enquête, vous devrez donner votre consentement (en cochant la case prévue à cet effet) concernant le texte d'informations détaillées et de consentement. L'enquête est composée de deux parties. La première partie inclut des questions sur votre utilisation et expérience (ou non) de la téléconsultation. La deuxième partie du questionnaire comprend quelques questions sociodémographiques. A la fin du questionnaire, vous retrouverez les informations de contact (e-mail) de l'investigatrice et du représentant académique afin de pouvoir poser vos questions et/ou recevoir davantage d'informations. La passation de l'enquête en ligne durera entre 10 à 15 minutes. **Risques et inconvénients** Comme dans toute étude, certains éléments relatifs à la participation pourraient engendrer d'éventuels affects négatifs temporaires, un inconfort passager, et/ou des pensées répétitives temporaires. De plus, certaines personnes pourraient ressentir un certain inconfort face à l'utilisation de la technologie virtuelle (i.e., passation d'un questionnaire en ligne) et/ou de la frustration en cas de problèmes techniques ou autres. **Bénéfices** Vous ne retirerez aucun avantage direct en participant à cette étude. Néanmoins, il se pourrait que vous, et d'autres personnes, puissent bénéficier de cette étude car ses résultats pourraient aider à informer la pratique clinique sur les besoins, questions et préoccupations concernant la téléconsultation. **Participation volontaire** Votre participation est volontaire et doit rester libre de toute contrainte estimée de votre part. La participation nécessitera votre accord en cochant la case de consentement à cet effet avant le début de l'enquête en ligne. Même après avoir coché votre accord, vous avez le droit d'arrêter votre participation à tout moment, sans donner d'explication.

INFORMATIONS COMPLÉMENTAIRES - sur la protection et les droits du participant à une étude **Comité d'éthique** Cette enquête a été évaluée et approuvée le 17 Juin 2020 par le Comité d'Ethique IPSY, qui permet d'assurer que

vos droits de participation à une étude sont respectés et protégés ; que les informations concernant l'étude ont été correctement et clairement communiquées, ainsi que les risques éventuels ; que ces derniers seront raisonnablement maitrisés ; et que l'enquête est scientifiquement adéquate et éthique. Néanmoins, l'approbation du Comité d'Ethique ne doit en aucun cas être perçue comme une incitation à participer à cette étude. **Dédommagements prévus pour votre participation** Aucune compensation n'est prévue pour la participation à cette enquête. **Garantie de confidentialité** Aucune information, permettant de vous identifier personnellement, ne vous sera demandée lors de la passation de l'enquête. L'enquête assure l'anonymat et respecte la confidentialité des données. En effet, les données seront gardées confidentielles conformément à la loi en vigueur du 30 juillet 2008 relative à la protection de la vie privée et à la réglementation européenne en vigueur du 25 mai 2018 (sur la protection des données à caractère personnel - Règlement Général sur la Protection des Données (RGPD)). Ceci veut dire que vous avez le droit de demander à l'investigatrice (et/ou représentant académique) la nature des données récoltées ainsi que leur utilité pour l'enquête. De plus, vous avez le droit de contacter l'investigatrice (et/ou représentant académique) afin de modifier vos réponses si vous jugez qu'elles seraient erronées. En participant à cette étude vous acceptez que vos données soient codées et analysées à des fins de recherches et de publications scientifiques. Il est important de noter que les résultats publiés aux travers de publications scientifiques et/ou autres ne permettront jamais de vous identifier personnellement. Seul l'équipe de recherche aura accès à vos données, mais aucune donnée ne permettra des vous identifier personnellement. Vos données seront conservées sur l'ordinateur de l'investigatrice, protégé par un mot de passe. Ces données seront effacées après la publication des résultats, sous forme de publications scientifiques. Pour toute question concernant la gestion de vos données personnelles, vous pouvez prendre contact à l'adresse suivante : privacy@uclouvain.be. En cas de plainte concernant le mode de traitement de vos données vous pouvez contacter l'autorité Belge de contrôle chargée de veiller au respect de la législation sur la protection des données.

CONSENT **CONSENTEMENT ÉCLAIRÉ**

En cochant la case mentionnant que vous avez lu le document d'information et de consentement, et que vous donnez votre accord de participation à l'enquête, vous soussignez : Avoir lu les informations relatives à l'étude et donner votre consentement libre et éclairé pour participer à l'enquête en ligne visant à récolter des informations sur votre utilisation et expérience de la téléconsultation dans le contexte de la pandémie du Coronavirus COVID-19. Avoir reçu ce document de consentement libre et éclairé et l'approuver (en cochant la case à cet effet) avant la passation en ligne. Vous déclarez avoir été renseigné·e sur la nature de l'enquête, ses objectifs, sa durée, ses éventuels avantages et désagréments et vous savez ce qui est attendu de vous. Vous vous êtes familiarisé.e avec le texte d'information et consentement éclairé. Avoir eu suffisamment le temps et la possibilité de réfléchir, poser et d'exprimer toutes vos questions et préoccupations concernant l'enquête ; qui ont reçu une réponse satisfaisante. Être au courant que cette enquête a été déposée et approuvée par le Comité d'Ethique IPSY. Avoir compris que votre participation à cette enquête est entièrement volontaire et que vous êtes dans le droit d'arrêter votre participation à tout moment sans devoir en justifier les raisons, ni en subir de conséquences.

Avoir compris que les données personnelles seront récoltées durant la passation de cette enquête en ligne, mais ne permettront pas de vous identifier personnellement et que l'équipe de recherche garantit la confidentialité et protection de ces données. Être d'accord au traitement confidentiel et protégé de vos données personnelles. En approuvant ce document (en cochant la case ci-dessous prévue à cet effet), vous : acceptez que vos données soient utilisées dans le respect de la loi belge du 30 juillet 2018 relative à la protection de la vie privée ; confirmez n'avoir subi aucune pression physique ni psychologique indue pour votre participation à l'enquête consentez de votre plein gré à participer à cette enquête. Je soussignée Jessica Notermans, étudiante de Master et investigatrice principale, garantis avoir fourni les informations nécessaires sur l'enquête et avoir fourni les informations détaillées de l'étude et le texte de consentement libre et éclairé aux participant·es. Je confirme qu'aucune pression n'a été exercée pour que le/la participant·e accepte de participer à l'enquête et que je suis prête et disponible à répondre à toutes questions et/ou inquiétudes supplémentaires, le cas échéant.

○ J'ai lu les informations relatives à l'étude et le texte de consentement et j'accepte de participer à cette enquête en ligne

End of Block: Présentation

Start of Block: START

Display This Question:

If CONSENTEMENT ÉCLAIRÉ En cochant la case mentionnant que vous avez lu le document d'information et... = J'ai lu les informations relatives à l'étude et le texte de consentement et j'accepte de participer à cette enquête en ligne

Q1 Depuis le confinement (16/03/2020), avez-vous proposé des prises en charge par téléconsultation ?

○ Oui

○ Non

End of Block: START

Start of Block: Thérapeute A

Display This Question:

If Depuis le confinement (16/03/2020),avez-vous proposé des prises en charges par t = Oui

Q2 Comparativement à la même période en 2019, le nombre de prises en charge entre Mars et Juin a (exprimé en % de différence):

Même si vous souhaitez répondre "0", cliquez sur le curseur avec la souris pour valider votre réponse. Ce curseur deviendra bleu une fois qu'il sera validé.

	Diminué de	N'a pas changé	Augmenté de
	-100 -80 -60 -40 -20	0	20 40 60 80 100
%	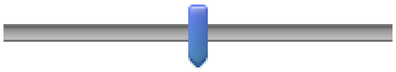		

Display This Question:

If Depuis le confinement (16/03/2020),avez-vous proposé des prises en charges par t = Oui

Q3 Comparativement à la même période en 2019, le nombre de prises en charge depuis Juin a (exprimé en % de différence):

Même si vous souhaitez répondre "0", cliquez sur le curseur avec la souris pour valider votre réponse. Ce curseur deviendra bleu une fois qu'il sera validé.

	Diminué de	N'a pas changé	Augmenté de
	-100 -80 -60 -40 -20	0	20 40 60 80 100
%	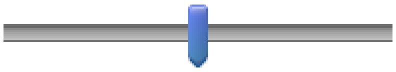		

Display This Question:

If Depuis le confinement (16/03/2020),avez-vous proposé des prises en charges par t = Oui

Q4 Avant votre première utilisation de la téléconsultation, quelle était votre représentation de celle-ci? Veuillez indiquer votre degré d'accord ou de désaccord avec les propositions suivantes. Veuillez préciser si nécessaire.

La téléconsultation limitera l'établissement d'une bonne relation thérapeutique	▼ Fortement en désaccord ... Tout à fait d'accord
Le manque d'information non-verbale sera trop important	▼ Fortement en désaccord ... Tout à fait d'accord
Il y aura trop de distractions chez la personne	▼ Fortement en désaccord ... Tout à fait d'accord
Je serai trop distrait.e	▼ Fortement en désaccord ... Tout à fait d'accord
Le/la client.e/patient.e ne sera pas assez engagé.e/présent.e	▼ Fortement en désaccord ... Tout à fait d'accord
Je ne serai pas assez engagé.e/présent.e	▼ Fortement en désaccord ... Tout à fait d'accord
La téléconsultation demande une bonne maitrise de l'outil informatique	▼ Fortement en désaccord ... Tout à fait d'accord
Les problèmes techniques auront un trop grand impact sur la communication	▼ Fortement en désaccord ... Tout à fait d'accord
Il sera difficile de mettre en place certaines interventions	▼ Fortement en désaccord ... Tout à fait d'accord
Mon infrastructure personnelle ne se prêtera pas à la téléconsultation (p. ex: manque d'intimité, pièce isolée pour la séance, etc.). Précisez ci-contre si nécessaire.	▼ Fortement en désaccord ... Tout à fait d'accord
La téléconsultation augmentera le nombre de dropout chez certaines personnes (p. ex: assuétudes). Précisez ci-contre si nécessaire.	▼ Fortement en désaccord ... Tout à fait d'accord

Display This Question:

If Depuis le confinement (16/03/2020),avez-vous proposé des prises en charges par t = Oui

Q5 Quel(s) média(s) utilisez-vous pour la téléconsultation ?

- ▢ Téléphone
- ▢ Chat (sans vidéo)
- ▢ E-mail
- ▢ Vidéoconférence

Display This Question:

If Quel(s) média(s) utilisez-vous pour la téléconsultation ? = Vidéoconférence

Q6 Quelle(s) plateforme(s) utilisez-vous pour les téléconsultations par vidéoconférence (p. ex: Skype, Zoom, Whereby)?

____

____

____

____

____

Display This Question:

If Depuis le confinement (16/03/2020),avez-vous proposé des prises en charges par t = Oui

Q7 Avant le confinement, avez-vous fait l'expérience de la téléconsultation en tant que:

- Aucune expérience
- Patient.e/Supervisé.e
- Superviseur.se
- Thérapeute

Display This Question:

If Depuis le confinement (16/03/2020),avez-vous proposé des prises en charges par t = Oui

Q8 Depuis le confinement, vous êtes-vous senti.e contraint.e à utiliser la téléconsultation?

- Pas du tout
- Légèrement
- Moyennement
- Fortement

Display This Question:

If Depuis le confinement (16/03/2020),avez-vous proposé des prises en charges par t = Oui

Q9 Vos collègues psychothérapeutes utilisent-ils/elles la téléconsultation?

○ Aucun

○ Très peu

○ Un certain nombre

○ La plupart

○ Tous

Display This Question:

If Depuis le confinement (16/03/2020),avez-vous proposé des prises en charges par t = Oui

Q10 Avez-vous eu des questions et/ou préoccupations concernant les aspects du Règlement Général sur la Protection des Données (RGPD) et autres aspects de déontologie à propos de(s) média(s) et/ou plateforme(s) virtuelle(s) utilisée(s) pour les téléconsultations?

○ Non

○ Oui, et j'ai trouvé des réponses satisfaisantes

○ Oui, mais il me reste des questions (Veuillez préciser) ____

Display This Question:

If Depuis le confinement (16/03/2020),avez-vous proposé des prises en charges par t = Oui

Q11 De manière générale, bénéficiez-vous d'un soutien (p.ex: informatique, collègues, superviseurs, et/ou autres) pour la mise en place des téléconsultations? Veuillez préciser le type de soutien.

◯ Aucun soutien

◯ Soutien léger (veuillez préciser ci-contre) ____

◯ Soutien moyen (veuillez préciser ci-contre) ____

◯ Soutien complet (veuillez préciser ci-contre) ____

Display This Question:

If Depuis le confinement (16/03/2020),avez-vous proposé des prises en charges par t = Oui

Q12 Avez-vous mis en place certaines actions afin d'encourager les personnes à entamer/continuer une prise en charge par téléconsultation?

◯ Oui

◯ Non

Display This Question:

If Avez-vous mis en place certaines actions afin d'encourager les personnes à entamer/continuer une... = Oui

Q13 Qu'avez-vous mis en place pour encourager les personnes à entamer/continuer une prise en charge par téléconsultation? Veuillez préciser si nécessaire.

☐ Communiquer avec le patient/client pour « faire le point » (par email, téléphone, ou autre). Précisez ci-contre si nécessaire. ____

☐ Donner des conseils généraux pour assurer que les conditions de téléconsultation soient optimales (p. ex: pièce au calme pour éviter les distractions et assurer l'intimité, assurer une bonne connexion internet, appareils chargés, etc.). Précisez ci-contre si nécessaire. ____

☐ Donner des informations sur l'efficacité des téléconsultations. Précisez ci-contre si nécessaire. ____

☐ Donner des informations sur l'utilisation de la plateforme virtuelle (ou autre média utilisé). Précisez ci-contre si nécessaire. ____

☐ Donner des informations sur la sécurité des données personnelles (confidentialité de la séance et du média utilisé). Précisez ci-contre si nécessaire. ____

☐ Faire un contact d'essai sur le média utilisé. Précisez ci-contre si nécessaire. ____

☐ Être flexible au niveau des ho**no**raires. Précisez ci-contre si nécessaire. ____

☐ Être flexible au niveau des ho**r**aires. Précisez ci-contre si nécessaire. ____

☐ Souligner l'importance du suivi psychothérapeutique pour le bien-être du patient/client. Précisez ci-contre si nécessaire. ____

☐ Autre(s) (veuillez préciser) ____

Display This Question:

If Depuis le confinement (16/03/2020),avez-vous proposé des prises en charges par t = Oui

Q14 Avez-vous dû adapter/aménager les téléconsultations pour certain(s) type(s) de population et/ou de problématique(s) de manière significative (p. ex: déroulement de la séance, choix de l'arrière plan, outils et procédures thérapeutiques, etc.)?

☐ Je n'ai pas dû adapter la téléconsultation de manière générale

☐ J'ai dû faire des changements mineurs (veuillez préciser ci-contre) ____

☐ J'ai dû faire des adaptations significatives en fonction du type de population

☐ J'ai dû faire des adaptations significatives en fonction du type de problématique

Display This Question:

If Avez-vous dû adapter/aménager les téléconsultations pour certain(s) type(s) de population et/ou d... = J'ai dû faire des adaptations significatives en fonction du type de population

Q15 Veuillez sélectionner la/les population(s) pour laquelle/lesquelles vous avez dû instaurer des changements et veuillez préciser ces changements (p. ex: déroulement de la séance, choix de l'arrière plan, outils et procédures thérapeutiques, etc.).

- ▢ Enfants ____
- ▢ Adolescents ____
- ▢ Adultes ____
- ▢ Personnes âgées ____
- ▢ Personnes en situation de handicap ____
- ▢ Personnes de cultures différentes ____
- ▢ Couple(s) ____
- ▢ Groupe(s)/Famille(s) ____
- ▢ Autre(s) (veuillez préciser) ____

Display This Question:

If Avez-vous dû adapter/aménager les téléconsultations pour certain(s) type(s) de population et/ou d... = J'ai dû faire des adaptations significatives en fonction du type de problématique

Q16 Veuillez sélectionner le(s) trouble(s) pour lequel/lesquels vous avez dû instaurer des changements et veuillez préciser les changements (p. ex: déroulement de la séance, choix de l'arrière plan, outils et procédures thérapeutiques, etc.).

- [ ] Troubles de l’humeur et apparentés ____
- [ ] Troubles anxieux et apparentés ____
- [ ] Troubles des conduites alimentaires ____
- [ ] Troubles neuro-développementaux ____
- [ ] Troubles sexuels ____
- [ ] Dépendance-addictions ____
- [ ] Troubles à symptômes somatiques (p.ex: somatoforme) et apparentés ____
- [ ] Troubles des conduites (comportements transgressifs) ____
- [ ] Troubles dissociatifs ____
- [ ] Troubles psychotiques ____
- [ ] Troubles de la personnalité ____

☐ Troubles non spécifiés par ailleurs ____

Display This Question:

If Depuis le confinement (16/03/2020),avez-vous proposé des prises en charges par t = Oui

Q17 De manière générale, quel est le pourcentage des personnes qui consultent pour:

Même si vous souhaitez répondre "0", cliquez sur le curseur avec la souris pour valider votre réponse. Ce curseur deviendra bleu une fois qu'il sera validé.

	0 10 20 30 40 50 60 70 80 90 100
Problème(s) déclenché(s) principalement par la crise COVID-19	
Problème(s) aggravé(s) significativement par la crise COVID-19	
Problème(s) indépendant(s) de la crise COVID-19	

Display This Question:

If Depuis le confinement (16/03/2020),avez-vous proposé des prises en charges par t = Oui

Q18 En comparaison aux consultations en face-à-face, à quel point la téléconsultation a-t-elle influencé (dégradé vs. amélioré) la relation thérapeutique au niveau de:

	Fortement dégradé	Légèrement dégradé	Pas de différence	Légèrement amélioré	Fortement amélioré
**Empathie** *(Comprendre et percevoir la réalité subjective du patient/client sans se laisser submerger par celle-ci ou se faisant influencer par ses propres préjugés et/ou valeurs)*	○	○	○	○	○
**Congruence** *(authenticité - capacité à identifier ses propres sentiments, pensées, et attitudes et à les exprimer au patient/client si jugé approprié)*	○	○	○	○	○
**Considération Positive Inconditionnelle** *(acceptation totale et inconditionnelle du patient/client sans jugement moral, éthique ou social)*	○	○	○	○	○
**Alliance thérapeutique**	○	○	○	○	○

Display This Question:

If Depuis le confinement (16/03/2020),avez-vous proposé des prises en charges par t = Oui

Q19 Avez-vous continué les prises en charge par téléconsultation après la période de confinement ?

○ Oui, à la demande du patient/client la téléconsultation restera une option

○ Oui, la téléconsultation deviendra majoritaire dans ma pratique clinique

○ Non

Display This Question:

If Depuis le confinement (16/03/2020),avez-vous proposé des prises en charges par t = Oui

Q20 Quelle est votre expérience personnelle concernant la téléconsultation, en comparaison au face-à-face ?

	Beaucoup moins bien	Légèrement moins bien	Pas de différence	Légèrement mieux	Beaucoup mieux
Efficacité thérapeutique	○	○	○	○	○
Satisfaction professionnelle	○	○	○	○	○
Epuisement, fatigue	○	○	○	○	○
Organisation, gestion du temps, des tâches, etc.	○	○	○	○	○
Facilité/Rapidité à recevoir le payement des honoraires	○	○	○	○	○

Display This Question:

If Depuis le confinement (16/03/2020),avez-vous proposé des prises en charges par t = Oui

Q21 Avez-vous d'autres remarques et/ou commentaires concernant la téléconsultation (amélioration possible au niveau des attitudes et outils thérapeutiques, choix de la plateforme, communication, informations partagées, expérience personnelle, représentation de la téléconsultation, etc.)?

____

End of Block: Thérapeute A

Start of Block: THERAPEUTE B

Display This Question:

If Depuis le confinement (16/03/2020),avez-vous proposé des prises en charges par t = Non

Q2B A quel point les éléments ci-dessous ont été déterminants dans la non poursuite des prises en charge par téléconsultation ? Veuillez préciser si nécessaire.

	Pas d'importance		Moyennement important		Très important
Ce mode de communication ne me paraît pas approprié pour une psychothérapie	○	○	○	○	○
J'ai des doutes concernant l'efficacité thérapeutique par téléconsultation	○	○	○	○	○
Les personnes ne souhaitaient pas entamer/continuer par téléconsultation	○	○	○	○	○
L'état des personnes ne nécessitait pas qu'une prise en charge continue	○	○	○	○	○
Raisons personnelles (p. ex : infrastructure limitée, enfants à charge, etc.). Précisez ci-contre si nécessaire.	○	○	○	○	○
Raisons financières (p.ex: pour bénéficier d'une aide). Précisez ci-contre si nécessaire.	○	○	○	○	○

Manque de soutien informatique	◯	◯	◯	◯	◯

Display This Question:

If Depuis le confinement (16/03/2020),avez-vous proposé des prises en charges par t = Non

Q3B Auriez-vous pu bénéficier d'un soutien (p.ex: informatique, de collègues, superviseurs, et/ou autres) dans la mise en place des téléconsultations? Veuillez préciser le type de soutien.

◯ Aucun soutien

◯ Soutien léger (veuillez préciser ci-contre) ____

◯ Soutien moyen (veuillez préciser ci-contre) ____

◯ Soutien complet (veuillez préciser ci-contre) ____

Display This Question:

If Depuis le confinement (16/03/2020),avez-vous proposé des prises en charges par t = Non

Q4B Avez-vous ressenti des contraintes (obligations) vous poussant à utiliser la téléconsultation?

◯ Pas du tout

◯ Légèrement

◯ Moyennement

◯ Fortement

Display This Question:

If Depuis le confinement (16/03/2020),avez-vous proposé des prises en charges par t = Non

Q5B Avant le confinement, vous avez eu de l'expérience de téléconsultation en tant que:

- ○ Aucune expérience
- ○ Patient.e/Supervisé.e
- ○ Superviseur.euse
- ○ Thérapeute

Display This Question:

If Depuis le confinement (16/03/2020),avez-vous proposé des prises en charges par t = Non

Q6B En tant que thérapeute, quelle est votre représentation de la téléconsultation? Veuillez indiquer votre degré d'accord ou de désaccord avec les propositions suivantes. Veuillez préciser si nécessaire.

La téléconsultation limite l'établissement d'une bonne relation thérapeutique	▼ Fortement en désaccord ... Fortement en accord
Le manque d'information non-verbale est trop important	▼ Fortement en désaccord ... Fortement en accord
Il y a trop de distractions chez le/la patient.e/client.e	▼ Fortement en désaccord ... Fortement en accord
Je serais trop distrait.e	▼ Fortement en désaccord ... Fortement en accord
Le/la client.e/patient.e n'est pas assez engagé.e/présent.e	▼ Fortement en désaccord ... Fortement en accord
Je ne serais pas assez engagé.e/présent.e	▼ Fortement en désaccord ... Fortement en accord
La téléconsultation demande une bonne maitrise de l'outil informatique	▼ Fortement en désaccord ... Fortement en accord
Les problèmes techniques ont un trop grand impact sur la communication	▼ Fortement en désaccord ... Fortement en accord
Il est difficile de mettre en place certaines interventions	▼ Fortement en désaccord ... Fortement en accord
Mon infrastructure personnelle ne se prête pas à la téléconsultation (p. ex: manque d'intimité, pièce isolée pour la séance, etc.). Précisez ci-contre si nécessaire.	▼ Fortement en désaccord ... Fortement en accord
La téléconsultation augmente le nombre de dropout pour certaines personnes (p. ex: assuétudes). Précisez ci-contre si nécessaire.	▼ Fortement en désaccord ... Fortement en accord

Display This Question:

If Depuis le confinement (16/03/2020),avez-vous proposé des prises en charges par t = Non

Q7B Vos collègues psychothérapeutes utilisent-ils/elles la téléconsultation?

- Aucun
- Très peu
- Un certain nombre
- La plupart
- Tous

Display This Question:

If Depuis le confinement (16/03/2020),avez-vous proposé des prises en charges par t = Non

Q8B Avez-vous l'intention d'utiliser la téléconsultation dans un futur relativement proche?

- Non
- Oui, si la pandémie persiste
- Oui, de toute manière

End of Block: THERAPEUTE B

Start of Block: Questionnaire démographique

QD1 **Veuillez compléter ces quelques questions portant sur vos données socio-démographiques**

Quel est votre genre?

- ◯ Féminin
- ◯ Masculin
- ◯ Autre (veuillez préciser) ____

QD2 Quel est votre niveau de formation de base?

- ◯ Enseignement supérieur de type court (Baccalauréat ou équivalent)
- ◯ Enseignement supérieur de type long (Master ou équivalent)
- ◯ Formation doctorale
- ◯ Autre(s) (veuillez préciser) ____

QD3 Quel est votre niveau de formation en psychothérapie?

- ○ Au moins une formation universitaire d'au moins 3 ans (DEA, Certificat d'Université, DU, ...)
- ○ Au moins une formation non universitaire d'au moins 3 ans
- ○ Plusieurs formations de moins de 3 ans
- ○ Une formation de moins de 3 ans
- ○ Autre(s) (veuillez préciser) ____

QD4 Quelle est votre orientation/formation psychothérapeutique?

- ▢ Dynamique
- ▢ Humaniste
- ▢ Psychanalytique
- ▢ Systémique
- ▢ Thérapie Cognitive-Comportementale (TCC)
- ▢ Intégrative
- ▢ Autre (veuillez préciser) ____

QD5 Quel est votre statut?

- ☐ Employé.e
- ☐ Indépendant.e
- ☐ Indépendant.e Complémentaire
- ☐ Autre(s) (veuillez préciser) ____

QD6 Expérience professionnelle. (veuillez préciser en nombre d'années par un chiffre)

____

QD7 Depuis juin 2020, quel est le pourcentage des consultations que vous faites en ligne?

Même si vous souhaitez répondre "0", cliquez sur le curseur avec la souris pour valider votre réponse. Ce curseur deviendra bleu une fois qu'il sera validé.

	0 25 50 75 100
Veuillez préciser	

QD8 Quelle est votre situation de (co)habitation?

- ◯ Je vis seul.e (avec ou sans enfants)
- ◯ Je vis en couple (avec ou sans enfants)
- ◯ Je vis avec d'autres personnes (colocataires, parents, etc.) (avec ou sans enfants)
- ◯ Autre situation (veuillez préciser) ____

QD9 Avez-vous des enfants qui vivent à votre domicile?

- ◯ Oui
- ◯ Non

Display This Question:

If Avez-vous des enfants qui vivent à votre domicile? = Oui

QD10 Combien d'enfants vivent à votre domicile? (précisez en chiffres)

____

Display This Question:

If Avez-vous des enfants qui vivent à votre domicile? = Oui

QD11 Veuillez préciser l'âge du plus jeune (p. ex: 9).

____

Display This Question:

If Avez-vous des enfants qui vivent à votre domicile? = Oui

QD11b Veuillez préciser l'âge du plus âgé (p. ex: 14).

____

Display This Question:

If Avez-vous des enfants qui vivent à votre domicile? = Oui

QD12 Dans la situation de confinement, la charge des enfants a-t-elle présenté des difficultés pour vos activités professionnelles?

- ○ Non
- ○ Oui, j'ai dû un peu réduire (de 10 à 30%) mes activités professionnelles pour m'occuper des enfants
- ○ Oui, j'ai dû réduire significativement (de 31 à 60%) mes activités professionnelles pour m'occuper des enfants
- ○ Oui, j'ai dû réduire fortement (de 61 à 80%) mes activités professionnelles pour m'occuper des enfants
- ○ Oui, j'ai dû extrêmement réduire (de 81 à 100%) voir arrêter mes activités professionnelles pour m'occuper des enfants

QD13 Quel est votre âge? (veuillez préciser en chiffre)

____

QD14 Quel est votre pays de résidence?

▼ Afghanistan ... Zimbabwe

End of Block: Questionnaire démographique

Appendix 2. Mean differences for attitudes towards teleconsultation between psychotherapists proposing and not proposing it (1: strongly disagree; 2: slightly disagree; 3: neither disagree nor agree; 4: slightly agree; 5 strongly agree)

Attitudes	Since the lockdown, have you proposed online consultation?	*Mean (M)*	*SD*	*t*	*p*
Teleconsultation will limit the development of a good therapeutic relationship	YES	3.79	1.190	-1.194	.065 [a]
	NO	4.08	.654		
The lack of non-verbal information will be too important	YES	3.89	1.112	-1.167	.244
	NO	4.17	0.917		
There will be too many distractions in the individual	YES	3.10	1.247	-1.642	.102
	NO	3.54	1.318		
I will be too distracted	YES	2.44	1.333	-0.75	.941
	NO	2.46	1.351		
The client/patient will not be engaged and present enough	YES	3.15	1.292	0.470	.642 [a]
	NO	3.04	1.083		
I will not be engaged/present enough	YES	2.44	1.295	-0.965	.335
	NO	2.71	1.429		
Teleconsultation requires good handling of informatic tools	YES	3.45	1.323	-1.523	.129
	NO	3.88	1.191		
	YES	3.72	1.216	-3.933	<.001 [a]

Technical issues will have too big of an impact on communication	NO	4.50	0.885		
It will be difficult to set up some interventions	YES	4.34	0.970	-.780	.436
	NO	4.50	0.590		
My personal infrastructure will not be adequate for online consultation (*e.g.,* limited infrastructure, isolated room for the session, etc.)	YES	2.47	1.394	-3.247	.001
	NO	3.46	1.641		
Teleconsultation will increase dropout number in certain individuals (*e.g.,* addictions)	YES	3.34	1.080	2.051	.047[a]
	NO	3.04	0.624		

[a] equal variance not assumed.

Appendix 3. Correlations between therapeutic relationship, personal experience, and percentage of teleconsultation

		Empathy	Congruence	Unconditional Positive Regard	Therapeutic Alliance	Therapeutic Efficacy	Professional Satisfaction	Strain, Fatigue	Organisation, time and task management, etc.	Ease and Rapidity to receive payments	Percentage of online consultation since June
Empathy	Pearson *r*	1	.389**	.411**	.417**	.378**	.303**	.231**	.107	.068	.059
	p		<.001	<.001	<.001	<.001	<.001	.001	.127	.332	.404
	N	207	207	207	207	206	206	206	206	206	205
Congruence	Pearson *r*	.389**	1	.229**	.394**	.413**	.273**	.019	.017	.047	.111
	p	<.001		.001	<.001	<.001	<.001	.788	.813	.507	.114
	N	207	207	207	207	206	206	206	206	206	205

Unconditional Positive Regard	Pearson *r*	.411**	.229**	1	.309**	.264**	.207**	.152*	.032	.051	.124
	p	<.001	.001		<.001	<.001	.003	.029	.649	.465	.075
	N	207	207	207	207	206	206	206	206	206	205
Therapeutic Alliance	Pearson *r*	.417**	.394**	.309**	1	.472**	.382**	.071	.013	.059	.073
	p	<.001	<.001	<.001		<.001	<.001	.311	.851	.400	.298
	N	207	207	207	207	206	206	206	206	206	205
Therapeutic Efficacy	Pearson *r*	.378**	.413**	.264**	.472**	1	.596**	.146*	.148*	.072	.343**
	p	<.001	<.001	<.001	<.001		<.001	.036	.034	.306	.000
	N	206	206	206	206	206	206	206	206	206	205
Professional Satisfaction	Pearson *r*	.303**	.273**	.207**	.382**	.596**	1	.438**	.352**	.155*	.356**
	p	<.001	<.001	.003	<.001	<.001		<.001	<.001	.027	<.001

	N	206	206	206	206	206	206	206	206	206	205
Strain, Fatigue	Pearson *r*	.231**	.019	.152*	.071	.146*	.438**	1	.403**	.161*	.125
	p	.001	.788	.029	.311	.036	<.001		<.001	.021	.075
	N	206	206	206	206	206	206	206	206	206	205
Organisation, time and task management, etc.	Pearson *r*	.107	.017	.032	.013	.148*	.352**	.403**	1	.157*	.085
	p	.127	.813	.649	.851	.034	<.001	<.001		.024	.224
	N	206	206	206	206	206	206	206	206	206	205
Ease and Rapidity to receive payments	Pearson *r*	.068	.047	.051	.059	.072	.155*	.161*	.157*	1	.020
	p	.332	.507	.465	.400	.306	.027	.021	.024		.779
	N	206	206	206	206	206	206	206	206	206	205
Percentage of online	Pearson *r*	.059	.111	.124	.073	.343**	.356**	.125	.085	.020	1

consultation	*p*	.404	.114	.075	.298	<.001	<.001	.075	.224	.779	
since June	N	205	205	205	205	205	205	205	205	205	229

**. Correlation is significant at the 0.01 level (2-tailed).

*. Correlation is significant at the 0.05 level (2-tailed).

Appendix 4. Number of participants using specific videoconferencing platforms (*n* = 209)

**Platform**	***n***
Skype	133
Zoom	89
WhatsApp	61
Whereby	55
Microsoft Teams	18
FaceTime	16
Facebook Messenger	10
Jitsi	6
Doctolib	6
Cisco Webex	4
Google Meet	3
ClickDoc	2
GoogleDuo	2
Discord	2
Work specific platform	2
Ubiclic	1
Google Hangouts	1
HUG@home	1
CareConnect	1
Lifesize	1
MediConsult	1

Aide-alcool.be	1
Tawk.to	1
Webcamconsult	1
HIN Video	1
Platform developed by doctoranytime	1

Appendix 5. Number of participants (*n*) who adapted teleconsultation according to the type of population and/or disorder

Type of Population	*n*	Type of Disorder	*n*
Adults	19	Anxiety disorders and others	13
Children	10	Mood disorders and others	9
Families	9	Personality disorders	6
Adolescents	8	Neurodevelopmental disorders	6
Couples	5	Eating disorders	5
Elderly	5	Dissociative disorders	5
Individuals from different cultures	3	Psychotic disorders	4
Individuals with disabilities	2	Conduct disorders (transgressive behaviours)	2
		Somatic disorders (*e.g.*, somatoform and others)	2
		Sexual disorders	2
		Addictive disorders	1
